# Evaluation of SARS-CoV-2 Spike Protein Antibody Titers in Cord Blood after COVID-19 Vaccination during Pregnancy in Polish Healthcare Workers: Preliminary Results

**DOI:** 10.3390/vaccines9060675

**Published:** 2021-06-19

**Authors:** Wojciech Zdanowski, Tomasz Waśniewski

**Affiliations:** 1 Department of Gynaecology and Obstetrics, Gynaecological Oncology Clinical Ward, Regional Specialist Hospital, ul. Żołnierska 18, 10-561 Olsztyn, Poland; tomasz.wasniewski@uwm.edu.pl; 2 Department of Obstetrics and Gynaecology, School of Medicine, Collegium Medicum, University of Warmia and Mazury, 10-561 Olsztyn, Poland

**Keywords:** COVID-19, pregnancy, vaccine

## Abstract

Background: The coronavirus disease 2019 (COVID-19) pandemic has given rise to the need to develop a vaccine as quickly as possible. As pregnant women are at increased risk of contracting severe COVID-19, with higher mortality, it is essential to assess the safety of the vaccines administered during pregnancy. Methods: The aim of this study was to determine the titer of specific maternal and cord antibodies against severe acute respiratory syndrome coronavirus 2 S protein after antenatal vaccination. The secondary objective was to evaluate the ratio of the umbilical cord to the maternal antibody titers. Patients included in the study were enrolled after undergoing voluntary vaccination against COVID-19 during pregnancy at different weeks of gestation. All patients analyzed in our initial study were vaccinated with the BNT162b2 mRNA COVID-19 vaccine. Results: The results of the current study document high anti-S total IgG antibody titers in cord serum at birth in all mother–infant pairs analyzed. The mean umbilical cord blood sample IgG antibody titer anti-S protein was 1026.51 U/mL (±SD 769.25). The mean cord-to-maternal anti–S IgG antibody ratio was 1.28 (±SD 0.798). A significant positive correlation was observed between the week of gestation at which the first dose was administered and the week of gestation at which the second dose was administered, and the respective cord-to-maternal ratio (r = 0.48; *p* = 0.0029) for the first dose and (r = 0.39; *p* = 0.0102) for the second dose. Conclusions: To date, despite the prevalence of COVID-19 vaccination, there is a lack of conclusive evidence supporting the safety and efficacy of vaccination of pregnant women. Therefore, the results we present are complementary. Our study suggests that maternal immunization may provide neonatal protection through the transplacental transfer of antibodies. Of particular importance is the demonstration that antibody transfer is correlated with the time from vaccination to delivery, which may allow future determination of the optimal timing of COVID-19 vaccination in pregnant women.

## 1. Introduction

The coronavirus disease 2019 (COVID-19) pandemic has become an indefinite global public health crisis. The elevated vulnerability of women during pregnancy, as well as experiences from previous coronavirus outbreaks, have heightened concerns around maternal and fetal complications [1]. Pregnant women are at an increased risk of developing severe COVID-19, with higher mortality rates, compared to non-pregnant women. The efficacy and safety of vaccines for use in pregnant women, fetuses, and infants remain undefined. Pregnant and lactating women have been excluded from clinical trials of existing COVID-19 vaccines [2,3,4]. According to the recommendations issued by the American College of Obstetricians and Gynecologists, the Center for Disease Control and Prevention, and the Royal College of Obstetricians and Gynecologists, COVID-19 vaccination should not be withheld in pregnant patients. Moreover, vaccination should primarily be considered in pregnant women with occupational exposure, such as healthcare workers. In Poland, the distribution of vaccines against COVID-19 was initially offered to priority groups, including medical personnel [5,6,7]. Consequently, there is urgent need for recommendations on whether pregnant women should or should not receive a COVID-19 vaccine. 

Antibodies, such as IgM and IgG, are among the primary mechanisms of the immune response to SARS-CoV-2 infection [8]. IgG antibodies produced after an intragestational vaccination with other vaccines, such as DTP or influenza, cross the placenta and provide innate passive immunity in children up to three months after birth [9].

Coronavirus genomes encode four main structural proteins: spike (S), membrane (M), envelope (E), and nucleocapsid (N). The S protein forms the characteristic superficial spikes of coronaviruses. Each monomer of protein S consists of an N-terminal S1 subunit and a membrane-bound S2 subunit to form a receptor-binding domain (RBD) [10,11,12,13]. Following BNT162b2 mRNA COVID-19 vaccination, the serum concentration of RBD-binding immunoglobulin increases with the dose of vaccine and after the second dose [14]. The aim of this preliminary study was to determine the titers of maternal and newborn-specific antibodies against SARS-CoV-2 S protein after antenatal vaccination. We also aimed to estimate the cord-to-maternal anti–S antibody ratio, measured by antibody titers against SARS-CoV-2 S protein.

## 2. Materials and Methods

### 2.1. Study Characteristics

**Figure 1:**
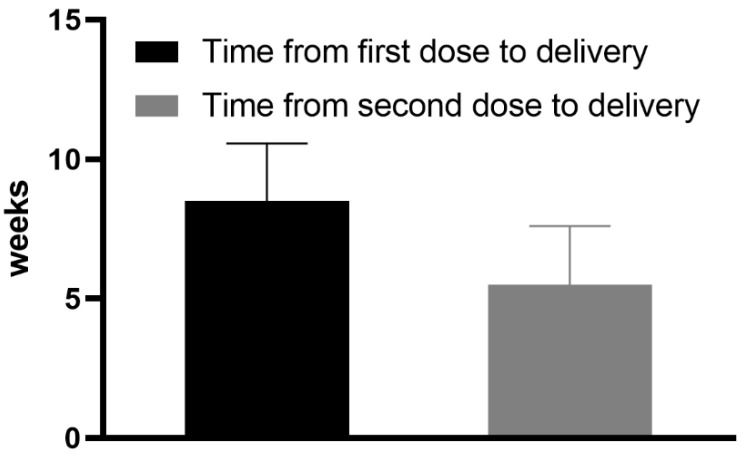
Mean time (weeks) from the first and the second dose of vaccine to delivery.

This was a retrospective, preliminary study. The present results were obtained using the blood and umbilical cord blood of 16 mothers on the day of delivery. All patients participating in this preliminary study were vaccinated with two doses of BNT162b2 mRNA COVID-19 vaccine between the 29th and 36th week of gestation; that is, the first dose was administered between the 29th and 36th week of gestation (the first dose) and the second dose between the 32nd and 40th week of pregnancy. The interval between vaccination and delivery is presented in Figure 1 and in Table 1.

Samples from all patients were collected on the day of delivery. Newborn blood samples were collected from the umbilical cord after clamping. Blood samples were collected by midwives after the instructional training. All the midwives had valid Polish professional licenses. The material was tested at the Provincial Specialist Hospital in Olsztyn. 

All the patients in the study had singleton pregnancies. The patients had voluntarily received vaccinations against COVID-19 during their pregnancies, at various weeks of gestation, and all were medical doctors with valid professional licenses in Poland. The vaccine was administered intramuscularly, according to the manufacturer’s protocol.

The study is still in progress and currently includes 150 female patients who had been vaccinated against COVID-19 during pregnancy. The study was approved by the Bioethics Committee of the Medical College of the University of Warmia and Mazury (approval no. 07/2021). Informed written consent was obtained from all the patients involved in the study. The inclusion criteria were age above 18 years and vaccination with two doses of COVID-19 vaccine during pregnancy. The exclusion criteria were as follows: COVID-19 confirmed with polymerase chain reaction (PCR) before or after COVID-19 vaccination, one or two doses of COVID-19 vaccination before pregnancy, and severe acquired or congenital immunodeficiency. Qualification of the study occurred after the vaccination. 

### 2.2. Laboratory Methods

In the present study, the in vitro qualitative and quantitative determination of total antibodies (IgG) developed against SARS-CoV-2 in human serum samples was performed using an electrochemiluminescence immunoassay.

The Elecsys^®^ Anti-SARS-CoV-2 assay (Roche Diagnostics, Basel, Switzerland) was used to detect the presence of antibodies to a recombinant protein that represents the nucleocapsid (N) antigen, whereas the concentration (U/mL) of antibodies to the SARS-CoV-2 spike (S) protein receptor binding domain (RBD) was analyzed using the Elecsys^®^ Anti-SARS-CoV-2-S-RBD assay (Roche Diagnostics, Switzerland). Both assays were performed according to the manufacturer’s instructions. The techniques used were based on a double-antigen sandwich reaction. The tested serum (20 µL) was incubated with biotinylated recombinant antigen specific for SARS-CoV-2 or SARS-CoV-2-S-RBD and recombinant antigen specific for SARS-CoV-2 or SARS-CoV-2-S-RBD labeled with ruthenium complex. Chemiluminescent emission was measured using a photomultiplier Cobas e immunoassay analyzer (Cobas e601, Roche Diagnostics, Switzerland). The results from Elecsys^®^ Anti SARS-CoV-2 were quantified using the software automatically by comparing the electrochemiluminescence signal generated from the sample reaction product with the cut-off level signal previously derived by calibration. A cut-off index of ≥1.0 U/mL was classified as reactive. In the Elecsys^®^ Anti-SARS-CoV-2-S-RBD assay, quantitative results were obtained from the calibration curve prepared for the analyzer, based on two-point calibration and the calibration curve. A concentration of <0.80 U/mL was considered negative and ≥0.80 U/mL was considered positive. The specificity and sensitivity of the methods were approximately 99% [15,16].

### 2.3. Collection of Variables

The following data were obtained from the medical records: the mother’s date of birth, last menstrual period date, parity, history of immunodeficiency disorders, dates of COVID-19 vaccine administration, blood drawing dates, newborn’s Apgar scores at 0, 5, and 10 min, newborn’s birth date, sex, and birth weight. Maternal and cord blood anti-S antibody levels were determined as dependent variables.

### 2.4. Statistical Analysis

The data were analyzed using the Statistica software (version 13.3, StatSoft, Kraków, Poland). Quantitative data are presented as mean ± standard deviation (SD). Data expressed on a qualitative scale were presented as the number and percentage of the sample. Pearson’s or Spearman’s correlation analysis was used to assess compliance with normal distribution. The results were considered statistically significant at *p* < 0.05.

## 3. Results

### 3.1. Main Characteristics of the Studied Population

Blood samples were collected and analyzed from 16 mothers on the day of delivery and from the umbilical cord of 16 newborns. The maternal and neonatal demographic and clinical data taken into consideration in the analysis are presented in Table 1.

### 3.2. Characteristics of Results

**Figure 2:**
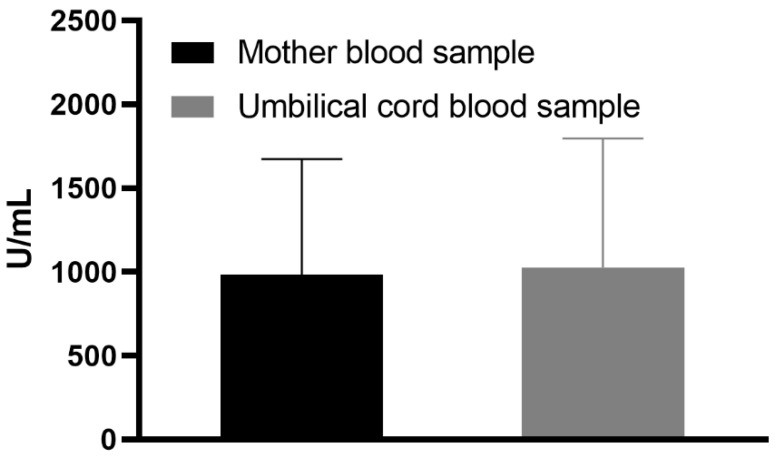
Mean anti-S antibody titers in maternal and umbilical cord blood.

It is important to note that no mothers had severe pregnancy or neonatal complications. None of the maternal blood and cord blood samples tested had specific SARS-CoV-2 anti-nucleocapsid antibody titers above the cut-off level (>1.0 U/mL). Antibodies against the SARS-CoV-2 S protein were identified in 100% (n = 16) of the tested maternal and in 100% (*n* = 16) of umbilical cord blood samples. All the samples had titers over 0.8 U/mL. The mean maternal blood sample antibody titer against SARS-CoV-2 S protein was 984.37 U/mL (±689.4). The mean umbilical cord blood sample antibody titer against SARS-CoV-2 S protein was 1026.51 U/mL (±769.25), as shown in Figure 2. The mean cord-to-maternal anti–S antibody ratio was 1.28 ± 0.798. The mean interval between the first and second vaccine doses was 21.31 days (±1.85).

**Figure 3:**
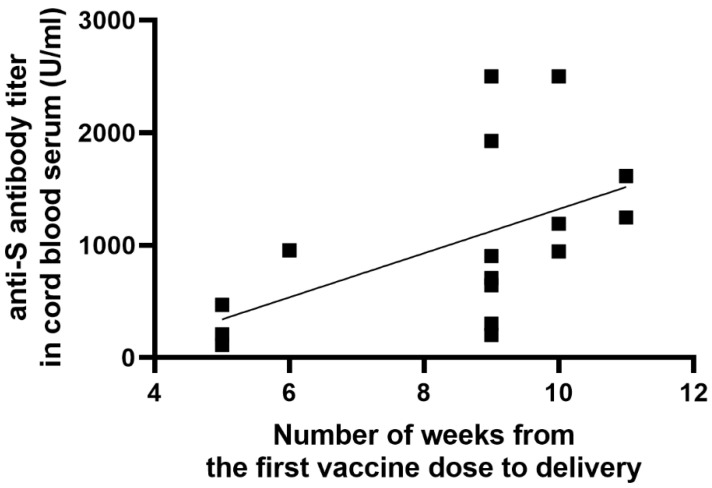
Correlation between the number of weeks from the first vaccine dose to delivery and the anti-S antibody titer in cord blood serum.

**Figure 4:**
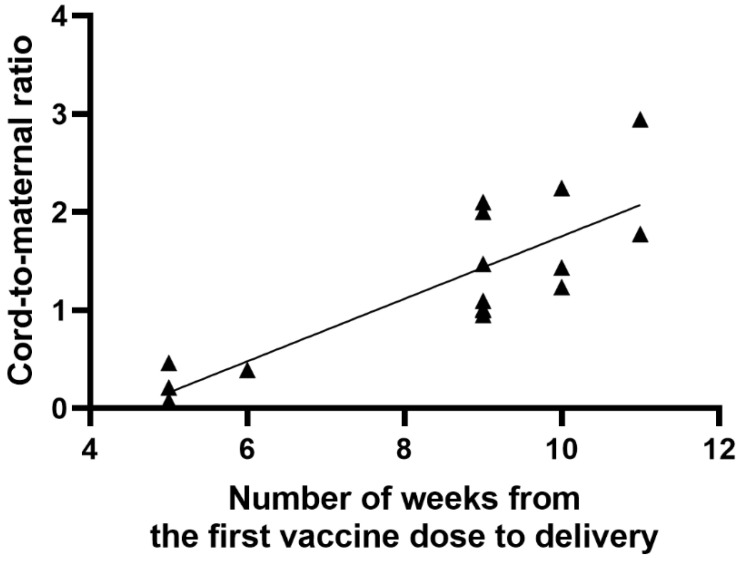
Correlation between the period (weeks) from the first vaccine dose to delivery and cord-to-maternal anti-S titer ratio.

**Figure 5:**
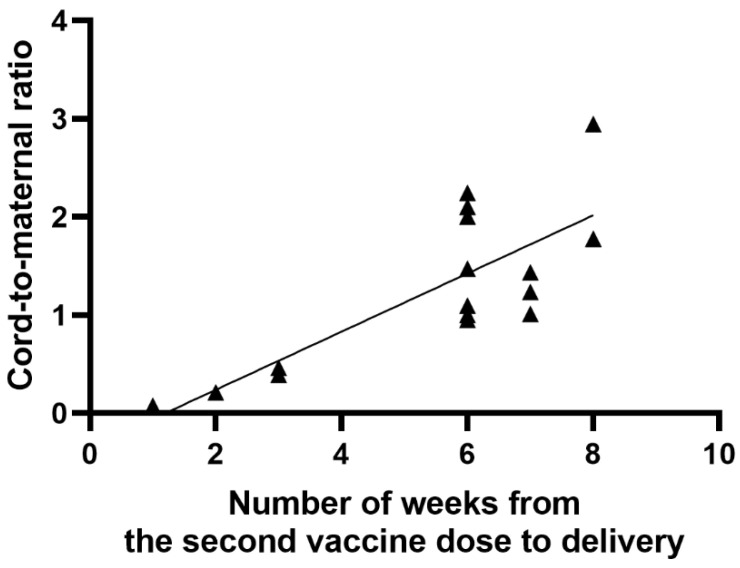
Correlation between the period (weeks) from the second vaccine dose to delivery and cord-to-maternal anti-S titer ratio.

**Figure 6:**
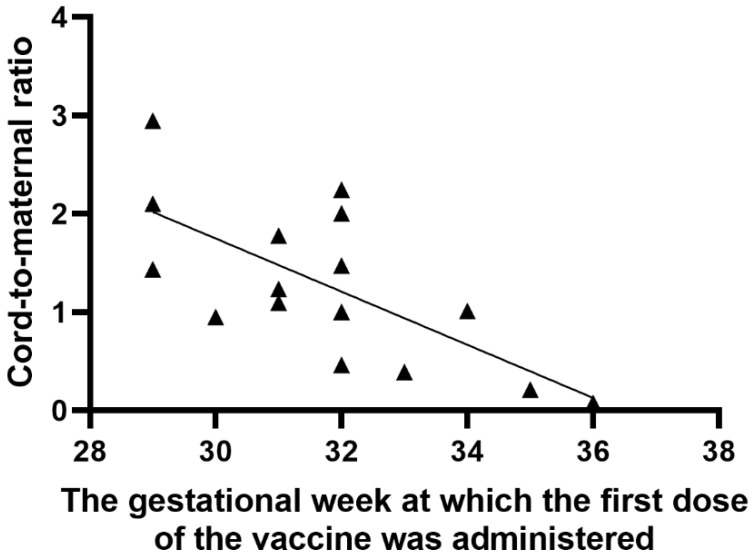
Correlation between the week of gestation when the first vaccine dose was administered and cord-to-maternal anti-S titer ratio.

**Figure 7:**
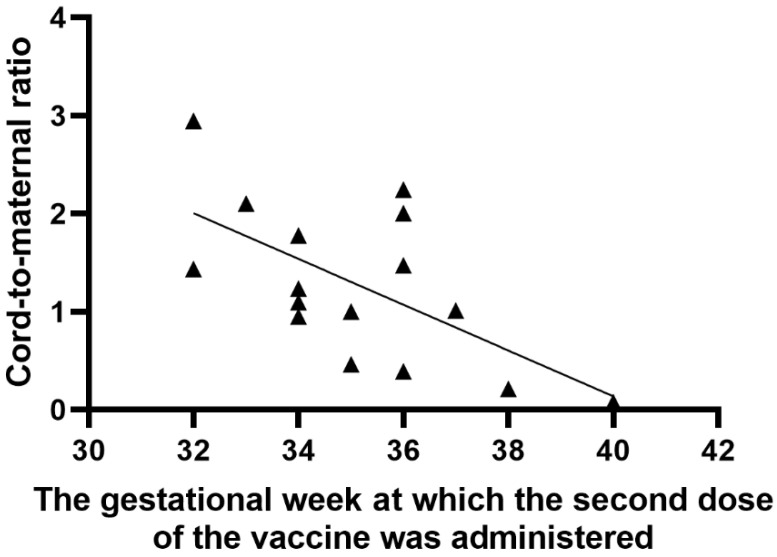
Correlation between the week of gestation when the second vaccine dose was administered and cord-to-maternal anti-S titer ratio.

A significant positive correlation was found between the number of weeks from the first vaccine dose to delivery and the anti-S antibody titer in cord blood serum (r = 0.63; *p* = 0.0092), as shown in Figure 3. Similarly, there was a significant positive correlation between the period (weeks) from the first and second vaccine dose to delivery and the umbilical cord-to-mother anti-S antibody ratio (r = 0.80; *p* = 0.0002) for the first dose and (r = 0.68; *p* = 0.0035) for the second dose, as shown in Figure 4 and Figure 5. It is important to note that the correlation coefficient of the number of weeks from the second dose to delivery was −0.50 for the maternal serum anti-S antibody titers and 0.45 for the cord blood serum antibody titers. Both were statistically insignificant (*p* = 0.0511 and *p* = 0.0774, respectively). A significant positive correlation was observed between the week of gestation at which the first dose was administered and the week of gestation at which the second dose was administered, and the respective cord-to-maternal ratio (r = 0.48; *p* = 0.0029) for the first dose and for the second dose (r = 0.39; *p* = 0.0102) (Figure 6 and Figure 7).

No significant variations were reported in anti-S antibody titers based on maternal age, body mass index (BMI), or the interval between vaccine doses.

## 4. Discussion

The results of the present study reveal high titers of anti-S antibodies in cord blood after birth, suggesting that maternal immunization may provide protection to newborns through the transplacental transfer of antibodies. The level of vaccine antibodies that provide immunity against COVID-19 in newborns has not yet been established and requires further investigation. The American College of Obstetricians and Gynecologists, the Centers for Disease Control and Prevention, and the Society for Maternal-Fetal Medicine published position statements supporting COVID-19 vaccine administration to pregnant individuals [5,6,8,17,18]. The Emergency Use Authorization information leaflets for health professionals for mRNA vaccines against SARS-CoV-2 indicate that there is insufficient evidence on the risks of the vaccines during pregnancy [19]. To date, safety, tolerability, and immune response data have been obtained in a double-blind phase I/II study of the COVID-19 BNT162b1 nucleoside lipid nanoparticle vaccine containing mRNA, which encodes the receptor binding domain (RBD) of SARS-CoV-2 virus marrow protein. Two injections of 1–50 μg of BNT162b1 induced intense antibody production, with RBD-binding IgG titers and CD4^+^ and CD8^+^ T-cell responses far exceeding those observed in the sera of individuals who recovered from COVID-19 [20]. The transfer of immune antibodies to the placenta depends on various factors [14]. The cord-to-maternal ratio of anti-S antibody titers after vaccination during pregnancy appears to be higher than the ratio of anti-receptor binding domain and anti-nucleocapsid antibodies after COVID-19 during pregnancy, as demonstrated in a study by Edlow AG et al. [21]. Cord serum samples from all newborns tested in our study showed the presence of specific antibodies against the S protein to SARS-CoV-2, and the same result was presented in study performed by Gray et al. [22]. According to a study by Vilajeliu et al. on the Tdap vaccine in the late second or third trimester of pregnancy, post-vaccination antibodies were found in 94% of newborns [23]. 

The trend in the correlation coefficients of the number of weeks from the first vaccine dose to delivery is worth noting. Maternal serum antibody titers were not significantly different; however, cord serum antibody titers differed significantly, as shown in Figure 3. This may be useful in the estimation of the optimal timing of vaccination during pregnancy. Grey et al. also suggested that the time since COVID-19 vaccination might be an essential factor determining transfer rates of specific IgG subclasses after maternal immunization [22]. The influenza vaccination is a good example of approach, which is commonly performed during pregnancy. Vaccination after the 29th week of gestation has been shown to be the most effective in conferring immunity to infants [24]. Based on the results obtained in our study, a correlation was observed between maternal serum anti-S antibody titers and newborn cord serum anti-S antibody titers. However, this difference was not statistically significant. It is also worth noting that even in a patient who received the second dose only seven days before delivery, a high titer of anti-S antibody was detected in the cord blood.

The limitations of our study are the small number of patients and the timing of the first and second doses of the vaccine being only in the third trimester of pregnancy. Further detailed observational and controlled studies are necessary to adequately assess the post-vaccination response in pregnant women and neonates. The results of this study may be relevant to public health. Our results may also be useful in the estimation of the risks and benefits of COVID-19 vaccination in pregnant women because the initial findings show no evident safety signals in pregnant subjects who had received COVID-19 mRNA vaccines [25].

## 5. Conclusions

Vaccinating pregnant women may be beneficial in protecting children in case of a risk of vaccine-preventable conditions in the first few months of life, as newborns are too young to receive vaccinations. The presence of antibodies against SARS-CoV-2 S protein in the cord blood may be the reason for passive immunity in newborns after vaccination during pregnancy. Of particular importance is the demonstration that antibody transfer is correlated with the time from vaccination to delivery, which may allow future determination of the optimal timing of COVID-19 vaccination in pregnant women. The determination of the presence and titers of specific antibody subclasses should be analyzed in future studies. There is need for the collection and systematic presentation of more data on the efficacy and safety of COVID-19 vaccination during pregnancy.

## Figures and Tables

**Table 1:** Maternal and newborn demographic and clinical data.

Variable	Included in the Analysis *n* = 16
Age of mothers (years)	31.2 * (±2.2 **) Min 28 Max 35
Parity	11 (69% ***)
≥1	
BMI	23.78 * (±4.51 **) Min 17.84 Max 34.95
Weeks of gestation at the first dose of COVID-19 vaccination (weeks)	31.75 * (±2.05 **) Min 29 Max 36
Weeks of gestation at the second dose of COVID-19 vaccination (weeks)	35.13 * (±2.13 **) Min 32 Max 40
Interval between the second dose of COVID-19 vaccination and collection of blood samples (day of delivery) (weeks)	5.5 * (±2.1 **) Min 1 Max 8
Interval between the first dose of COVID-19 vaccination and the collection of blood samples (day of delivery) (weeks)	8.5 * (±2.07 **) Min 5 Max 11
Weeks of gestation at delivery (weeks)	39.69 * (±1.01 **) Min 38 Max 41
Sex of newborn	
Male	8 (50% ***)
Female	8 (50% ***)
Weight of newborn (g)	3353 * (±495 **) Min 2270 Max 4000

BMI: body mass index. Min: minimum. Max: maximum. * Mean. ** Standard Deviation (±SD). *** Percentage of all surveyed patients.

## Data Availability

Paper data are available for review upon request.

## References

[1] Masmejan S., Pomar L., Lepigeon K., Favre G., Baud D., Rieder W. (2020). COVID-19 et grossesse [COVID-19 and pregnancy]. Rev. Med. Suisse.

[2] Rasmussen S.A., Kelley C.F., Horton J.P., Jamieson D.J. (2021). Coronavirus disease 2019 (COVID-19) vaccines and pregnancy: What obstetricians need to know. Obstet. Gynecol..

[3] European Medicines Agency Procedure No. EMEA/H/C/005735/0000. Assessment Report: COVID-19 mRNA Vaccine (Nucleoside-Modified). https://www.ema.europa.eu/en/documents/assessment-report/comirnaty-epar-public-assessment-report_en.pdf.

[4] Stafford I.A., Parchem J.G., Sibai B.M. (2021). The coronavirus disease 2019 vaccine in pregnancy: Risks, benefits, and recommendations. Am. J. Obstet. Gynecol..

[5] COVID-19 (Coronavirus Disease): People with Certain Medical Conditions. https://www.cdc.gov/coronavirus/2019-ncov/need-extra-precautions/people-with-medical-conditions.html.

[6] COVID-19 (Coronavirus Disease): Information about COVID-19 Vaccines for People Who Are Pregnant or Breastfeeding. https://www.cdc.gov/coronavirus/2019-ncov/vaccines/recommendations/pregnancy.html.

[7] Royal College of Obstetricians and Gynaecologists Updated Advice on COVID-19 Vaccination in Pregnancy and Women Who Are Breastfeeding. https://www.rcog.org.uk/en/news/updated-advice-on-covid-19-vaccination-in-pregnancy-and-women-who-are-breastfeeding/.

[8] Roberts J.N., Gruber M.F. (2015). Regulatory considerations in the clinical development of vaccines indicated for use during pregnancy. Vaccine.

[9] Abu Raya B., Edwards K.M., Scheifele D.W., Halperin S.A. (2017). Pertussis and influenza immunisation during pregnancy: A landscape review. Lancet Infect. Dis..

[10] Letko M., Marzi A., Munster V. (2020). Functional assessment of cell entry and receptor usage for SARS-CoV-2 and other lineage B betacoronaviruses. Nat. Microbiol..

[11] Wrapp D., Wang N., Corbett K.S., Goldsmith J.A., Hsieh C.-L., Abiona O., Graham B.S., McLellan J.S. (2020). Cryo-EM structure of the 2019-nCoV spike in the prefusion conformation. Science.

[12] Xu H., Zhong L., Deng J., Peng J., Dan H., Zeng X., Li T., Chen Q. (2020). High expression of ACE2 receptor of 2019-nCoV on the epithelial cells of oral mucosa. Int. J. Oral Sci..

[13] Hoffmann M., Kleine-Weber H., Schroeder S., Krüger N., Herrler T., Erichsen S., Schiergens T.S., Herrler G., Wu N.-H., Nitsche A. (2020). SARS-CoV-2 cell entry depends on ACE2 and TMPRSS2 and is blocked by a clinically proven protease inhibitor. Cell.

[14] Sahin U., Muik A., Derhovanessian E., Vogler I., Kranz L.M., Vormehr M., Baum A., Pascal K., Quandt J., Maurus D. (2020). COVID-19 vaccine BNT162b1 elicits human antibody and Th1 T cell responses. Nature.

[15] L’Huillier A.G., Meyer B., Andrey D.O., Arm-Vernez I., Baggio S., Didierlaurent A., Eberhardt C.S., Eckerle I., Grasset-Salomon C., Huttner A. (2021). Antibody persistence in the first 6 months following SARS-CoV-2 infection among hospital workers: A prospective longitudinal study. Clin. Microbiol. Infect..

[16] Higgins V., Fabros A., Kulasingam V. (2021). Quantitative measurement of anti-SARS-CoV-2 antibodies: Analytical and clinical evaluation. J. Clin. Microbiol..

[17] The American College of Obstetricians and Gynecologists (2020). Vaccinating Pregnant and Lactating Patients against COVID-19. https://www.acog.org/en/clinical/clinical-guidance/practice-advisory/articles/2020/12/vaccinating-Pregnant-and-Lactating-Patients-Against-COVID-19.

[18] Society for Maternal-Fetal Medicine (SMFM) Statement SARS-Co-V-2 Vaccination in Pregnancy. https://s3.amazonaws.com/cdn.smfm.org/media/2591/SMFM_Vaccine_Statement_12-1-20.

[19] Klein S.L., Creisher P.S., Burd I. (2021). COVID-19 vaccine testing in pregnant females is necessary. J. Clin. Investig..

[20] Esposito S., Bosis S., Morlacchi L., Baggi E., Sabatini C., Principi N. (2012). Can infants be protected by means of maternal vaccination?. Clin. Microbiol. Infect..

[21] Edlow A.G., Li J.Z., Collier A.-R.Y., Atyeo C., James K.E., Boatin A.A., Gray K.J., Bordt E.A., Shook L.L., Yonker L.M. (2020). Assessment of maternal and neonatal SARS-CoV-2 viral load, transplacental antibody transfer, and placental pathology in pregnancies during the COVID-19 pandemic. JAMA Netw. Open.

[22] Vilajeliu A., Goncé A., López M., Costa J., Rocamora L., Ríos J., Teixidó I., Bayas J.M. (2015). Combined tetanus-diphtheria and pertussis vaccine during pregnancy: Transfer of maternal pertussis antibodies to the newborn. Vaccine.

[23] Gray K.J., Bordt E.A., Atyeo C., Deriso E., Akinwunmi B., Young N., Baez A.M., Shook L.L., Cvrk D., James K. (2021). Coronavirus disease 2019 vaccine response in pregnant and lactating women: A cohort study. Am. J. Obstet. Gynecol..

[24] Omer S.B., Clark D.R., Madhi S.A., Tapia M.D., Nunes M.C., Cutland C.L., Simões E.A.F., Aqil A.R., Katz J., Tielsch J.M. (2020). Efficacy, duration of protection, birth outcomes, and infant growth associated with influenza trials vaccination in pregnancy: A pooled analysis of three randomized controlled. Lancet Respir. Med..

[25] Shimabukuro T.T., Kim S.Y., Myers T.R., Moro P.L., Oduyebo T., Panagiotakopoulos L., Marquez P.L., Olson C.K., Liu R., Chang K.T. (2021). Preliminary findings of mRNA Covid-19 vaccine safety in pregnant persons. N. Engl. J. Med..

